# A Pilot Study on Effects of Acupuncture and Moxibustion by Hyperspectral Imaging Technique

**DOI:** 10.1155/2014/135212

**Published:** 2014-12-03

**Authors:** Dong Zhang, Yin-long Li, Shu-you Wang, Xiao-dong Bai, Xiao-jing Song, Shun-yue Li

**Affiliations:** Institute of Acupuncture and Moxibustion, China Academy of Chinese Medical Sciences, No. 16 Nanxiaojie Dongzhimen, Beijing 100700, China

## Abstract

This study was to observe the effects of acupuncture and moxibustion on spectrum features of acupoint using hyperspectral imaging (HSI) technique. HSI of the Neiguan (PC6) in the acupuncture groups, moxibustion groups, and control groups was scanned by the hyperspectral imager to analyze the spectrum features and the variations within the wavelength of 400–1000 nm and explore the relationship between the spectral characteristics and effects of acupuncture and moxibustion. The light absorption intensity was slightly reduced within the wave band of 540–590 nm after acupuncture. The absorption intensity of PC6 before moxibustion was significantly higher than that after moxibustion, and the maximum reduction was found at the wavelength of 580 nm with 20.5% reduction, *P* < 0.05. There was no significant change of the spectrum of palm and PC6 and the spectrum curves of the acupoint were basically identical in control group. The light absorption intensity of PC6 of human body was weakened after Acu-mox. Specific wavelengths were all exhibited at 580 nm and the effect of moxibustion was more significant. HSI technique can be used to measure the spectral characteristics of the acupoint areas. This first time research would be significant and beneficial for study on the effect of acupuncture and moxibustion.

## 1. Introduction 

Hyperspectral imaging (HSI) technique is an image data technique which has been developed in the recent two decades on the basis of spectrum, imaging, and computer image processing. It has integrated the advanced technologies from the fields of optics, optoelectronics, electronics, information processing, computer science, and so on. It is an emerging technology with organic integration of traditional two-dimension imaging technology and spectrum technology [1]. HSI technique is an image data technique which is continuous imaging of the object within the wavelengths from visible light to near-infrared on the basis of the multispectral imaging. Spectral information will also be obtained when the space imaging information of the target or sample is obtained; in other words, the three-dimensional data with image-spectrum merging is obtained [2, 3]. The features of this technique include a multiwavelength (from visible light to near-infrared), high spectral resolution (≤1 nm), a narrow bandwidth (≤10^−2^ *λ*), wide spectral range, and image-spectrum merging. The spectral analysis technique has been effectively combined with image analysis technique so that this technique can resolve both spectrum and image; spectral imaging technique can be used for the qualitative and quantitative analyses of the object being examined as well as for the positioning analysis of the object [4]. As a high-tech technique which can reveal the material characteristic information, hyperspectral imaging technique has been preliminarily applied in the medical diagnosis studies [5, 6]. Because application of hyperspectral imaging technique is just beginning in the medical science, there is no report of acupuncture, main and collateral channels and acupoint studies. In this paper, this technique is used as the study method to observe the spectral characteristics and the changes of the acupoint area after acupuncture. The spectroscopy was used to study the acupuncture effects for the first time and to explore the possibility and values of HSI technique in acupuncture research field.

## 2. Methods

### 2.1. Subjects

Ten healthy, without skin disease, adult volunteers (4 females and 6 males, ages ranged from 22 to 25 years) were recruited in this study. Prior to participation, all subjects provided written informed consent. All experimental procedures were approved by the Ethical Committee of China Academy of Medical Sciences and conducted in accordance with the international accepted principles.

### 2.2. Experimental Instruments

*Hyperspectral Imager.* Canada Photon hyperspectral imager was used for the study; the spectral wavelength range was 400–1000 nm and the spectral resolution was ≤1 nm. Cube imaging mode was adopted in this study to determine the size of collection scope according to the collection site. This instrument was connected to a computer. PHyspec image application software was used for processing, analysis, and saving of hyperspectral images.

### 2.3. Experimental Environment

The temperature of the detection environment was 25 ± 1°C, and the relative humidity was 30%–60%. The indoor and outdoor ventilation was isolated. There was no solar radiation in the room, the radiation sources were 4 × 150 W full spectrum halogen lamps, the radiation lamps were in two parallel lines, two lamps in each line, and the space was about 60 cm. The objects were under the direct radiation.

### 2.4. Acupuncture and Moxibustion Method

Subjects were assigned into control group (Con group), acupuncture group (Acu group), and moxibustion group (Mox group) in this experiment, and the subjects in different groups were the subjects from the same batch (10 persons). Hyperspectral image collection was conducted at the same time of different day in the same environment; the interval was 3 days for the same subject to receive acupuncture or moxibustion. 

*Con Group*. Hyperspectral images of the partial forearm and palmar surface including the PC6 of the subjects at 0 min and 10 min were collected. 

*Acu Group*. The 40 × 0.25 mm acupuncture needles (products of Suzhou Medical Instruments Co., Ltd.) were punctured into the unilateral PC6, and the depth of acupuncture was half an inch. The lifting-thrusting and twisting-rotating stimulation were done until both the practitioner and the volunteer felt the qi arrival; then the insertion was stopped, and the needle remained in place for 10 min. The hyperspectral images of the partial forearm and palmar surface including the PC6 of the subjects before and after acupuncture were collected. 

*Mox Group*. The moxibustion was conducted vertically above 2.5 cm of the PC6 with *φ*2 cm Hwato moxa sticks and it should be subjected to occurrence of flushing and tolerance of the subject. The hyperspectral images of the partial forearm and palmar surface including the PC6 point of the subjects before and after moxibustion were collected.

### 2.5. Scanning and Processing of Hyperspectral Images

1. The subjects waited quietly for 15 min in the detection environment and started the detection after they adapted to the environment with stable emotion. The forearms of the subjects should be placed in parallel on the fixed platform in front of the camera lens of the hyperspectral imager. Real-time imaging monitoring function of the PHySpec software was used to observe and select the optimal position; the parameters were as follows: the wavelength was 450–1000 nm, the interval was 10 nm, the exposure time was 1 s, the scanning background was black background, and the sources were 4 × 150 W halogen lamps. The images were scanned and saved by PHySpec software.
2. The standard white background cube was scanned. It was conducted with the parameters and sources similar to those described in (1).
3. Dark cube was scanned. It was conducted with the parameters and sources similar to those described in (1).
4. Cube normalization. A displayed cube was normalized by using a standard white background cube and a dark cube.
5. The pixel range of 40 mm × 40 mm for the acupoint in the cube was selected to display the spectrum curves and the value of reflecting intensity.

### 2.6. Analysis and Statistics

PHyspec software was used to intuitively analyze the cubes so as to learn the distribution characteristics of spectrum of PC6 on the forearm under normal condition and the spectral changes of PC6 after acupuncture and moxibustion. The spectrum curves of PC6 were extracted and displayed in each group, the changes were compared, and the spectrum characteristic was found; the light reflex intensity of each wavelength of the PC6 in each group was extracted according to the formula: rate of changes of reflecting intensity = [*a* − *b*/*b*] × 100%, in which *a* is intensity of 10 min and *b* is intensity of 0 min. SPSS17.0 statistical software was used to calculate the rates of change of light reflex intensity at different wavelength of each group,* t*-test was used to compare the difference between the rates of change of light reflex intensity at different wavelength in the same group, and *P* < 0.05 was considered as the statistical difference standard.

## 3. Results 

### 3.1. Intuitive Analysis of the Hyperspectral Images in Each Experimental Group

#### 3.1.1. Characteristics of the Hyperspectral Images of Forearm and Palm

**Figure 1:**
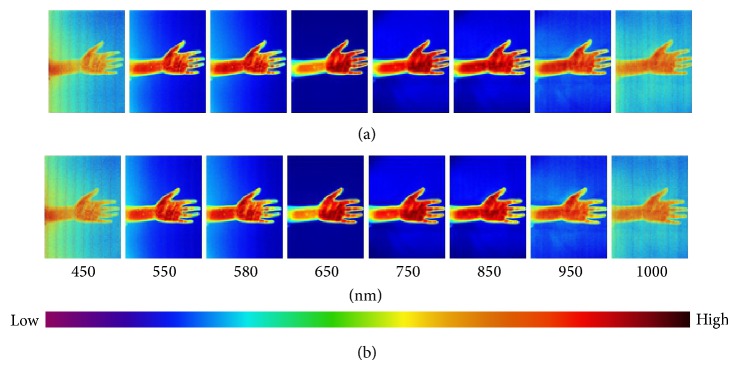
Hyperspectral images of the forearm and palm at different wavelengths at 0 and 10 min of the Con group. Note: the images of forearm and palm which are at normal condition at 0 min (a) and 10 min (b) are shown for selected wavelengths from 450, 550, 580, 650, 750, 850, 950, and 1000 nm. The color scale indicates the degree of light reflex intensity and the intensity from left to right is from weak to strong. The colors of the thenar eminence, antithenar eminence, and 2/3 part in the centre of the palm closely adjacent to antithenar eminence are darkest and the light reflex is strongest. The small part of the centre of the palm adjacent to thenar eminence is lighter and the light reflex is weaker. There is no significant change for the color distribution of the spectral images of PC6 and other areas on the hand between 0 min (a) and 10 min (b).

**Figure 2:**
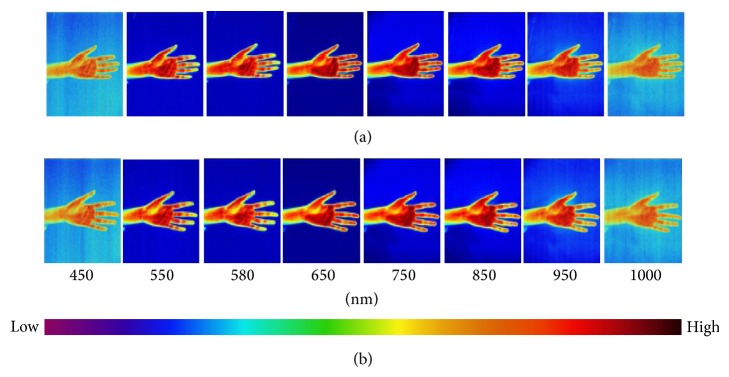
Hyperspectral images of the forearm and palm at different wavelengths at 0 and 10 min. Note: the images of forearm and palm which are at 0 min (a) and 10 min (b) of the Acu group are shown for selected wavelengths from 450, 550, 580, 650, 750, 850, 950, and 1000 nm. In (a), there is no significant change for the color distribution of the spectral images of PC6 and other areas on the hands compared to Figure 1(a). According to the color scale, in (b), there is left shift of color at the corresponding wavelength points compared to (a) which indicates that the light reflex intensity is weakened after acupuncture.

**Figure 3:**
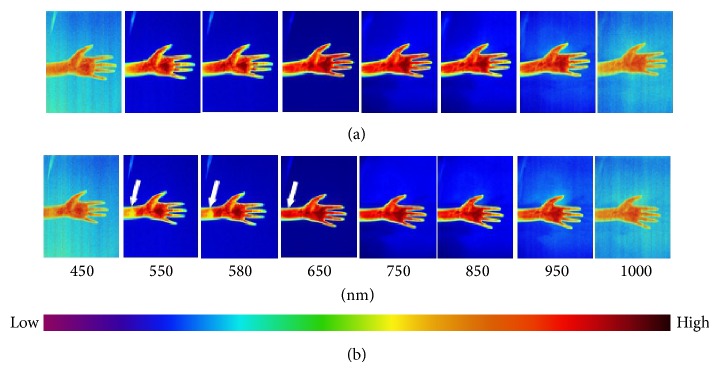
Hyperspectral images of the forearm and palm at different wavelengths at 0 and 10 min of the Mox group. The images of forearm and palm which are at 0 min (a) and 10 min (b) of the Mox group are shown for selected wavelengths from 450, 550, 580, 650, 750, 850, 950, and 1000 nm. In 3(a), there is no significant change for the color distribution of the spectral images of PC6 and other areas on the hands compared to Figure 1(a). According to the color scale, in 3(b), the left shift of color is significant at the corresponding wavelength points compared to 3(a) which indicates that the light reflex intensity is significantly weakened after moxibustion. The color change is most significant at the PC6 (indicated by the arrow) and colors of other areas of hands are also changed.

**Figure 4:**
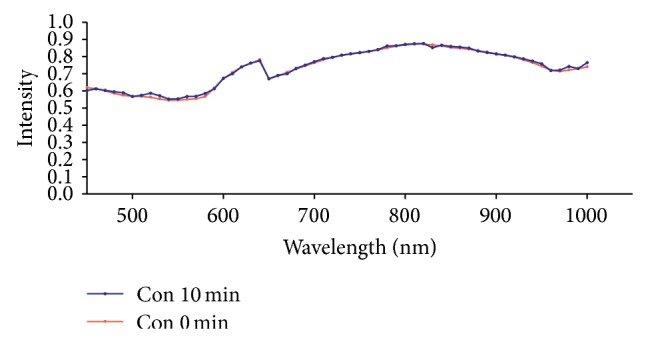
Comparison of the light reflex intensity values of PC6 before and after 10 min in the Con group (mean, *n* = 10). Note: the distribution mean values of curves at all the wavelengths at 0 min basically overlap those at 10 min which indicates that there is no significant change of light reflex intensity before and after 10 min.

**Figure 5:**
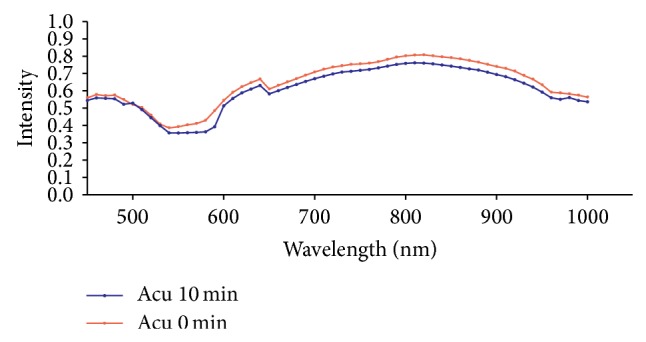
Comparison of the light reflex intensity values of PC6 before and after acupuncture in the Acu group (mean, *n* = 10). Note: the distribution mean values of curves at all the wavelength are different after 510 nm before and after acupuncture and the values after acupuncture are all lower than those before acupuncture which indicates that acupuncture leads to reduction of light reflex intensity.

**Figure 6:**
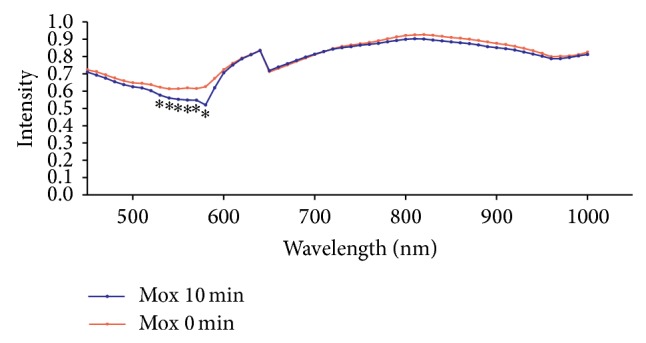
Comparison of the reflex intensity values of PC6 before and after moxibustion in the Mox group (mean, *n* = 10). Note: there is significant difference at wave band of 530–590 nm and wave band of 780–960 nm before and after the moxibustion; the values after moxibustion are lower than those before moxibustion which are especially significant at six points between 540 nm and 590 nm. There is statistical difference and ^*^ *P* < 0.05.

Fifty-six hyperspectral images in total have been collected for the forearm and palm at 450–1000 nm with the internal of 10 nm. Eight images of every 50 nm were displayed in Figures 1–6 in this paper. The color from left to right indicated that the light reflex intensity was from weak to strong according to the indication of the color scale. It can be seen from the images of each group before the stimulation that the color of the bilateral marginal area beside the inner side of the arm was light and the light reflex was relatively weaker; the color of the central line area of the arm was dark and the light reflex was relatively stronger. The color of most of the area of the palmar surface was light and the colors of the thenar eminence, antithenar eminence, and 2/3 part in the centre of the palm closely adjacent to antithenar eminence were darkest and the light reflex was strongest. The small part of the centre of the palm adjacent to thenar eminence was lighter and the light reflex was weaker; the color of the finger pulp was light and the light reflex was weak (Figures 1(a), 2(a), and 3(a)).

#### 3.1.2. Analysis of the Hyperspectral Image Change in Each Group

There was no significant change of color distribution of the hyperspectral images before and after 10 min in the Con group and the change at the PC6 was similar (Figure 1). There was color change of the images after acupuncture and moxibustion and the change was relatively smaller in the Acu group (Figure 2); the change in the Mox group was relatively large which was significant at PC6 area (indicated by the arrow). In Figure 3(b) (550 nm and 580 nm), the color significantly became lighter compared to Figure 3(a) (550 nm and 580 nm) which indicated the significant weakening of the light reflex intensity; the change of color distributions in other areas on the arm and palm was small (Figure 3).

### 3.2. Curve Characteristics Analysis of the Hyperspectral Images in Each Experiment Group

PHyspec image application software was used to display the spectrum curves of PC6 and the mean spectrum curves of the wavelength of 450–1000 nm at PC6 in each group were obtained. The spectrum curves at 0 min basically overlapped the spectrum curves at 10 min in the Con group which indicated that there was no significant change of light reflex intensity in absence of external stimulation and there was no statistical difference for this value in each wavelength, *P* > 0.05 (Figure 4). In the Acu group, the light reflex intensity was slightly reduced at the wave band of 570–590 nm after acupuncture for 10 min, but there was no statistical difference between the light reflect intensities before and after acupuncture in all the wavelengths, *P* > 0.05 (Figure 5). In the Mox group, the difference between the spectrum curves at wave band of 510–590 nm in the PC6 was great before and after moxibustion and there was obvious shift down after moxibustion which indicated the light reflex intensity was obviously weakened after moxibustion. There was significant statistical difference of light reflex intensity values at 7 wavelengths between 530 nm and 580 nm, *P* < 0.05. There was no significant change of spectrum curves at other wavelengths compared to those before moxibustion (Figure 6).

### 3.3. Analysis of the Rate of Light Reflex Intensity Change in Each Experiment Group

**Figure 7:**
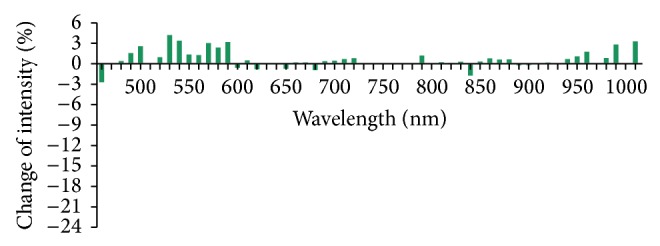
Comparison of rates of reflex intensity change at the PC6 after 10 min in the Con group (%, *n* = 10). Note: the rates of reflex intensity change of all the wavelengths are all lower than 5%.

**Figure 8:**
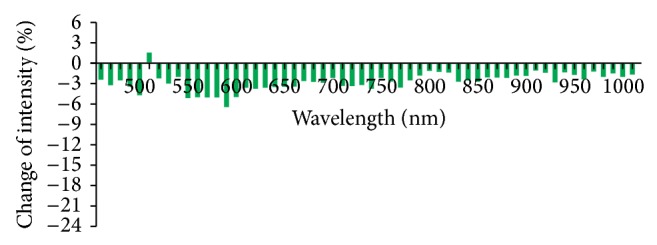
Comparison of rates of reflex intensity change at the PC6 after acupuncture in the Acu group (%, *n* = 10). Note: the rates of light reflex intensity change at all the wave bands are all negative. The reduction is greatest at 580 nm, −6.5%.

**Figure 9:**
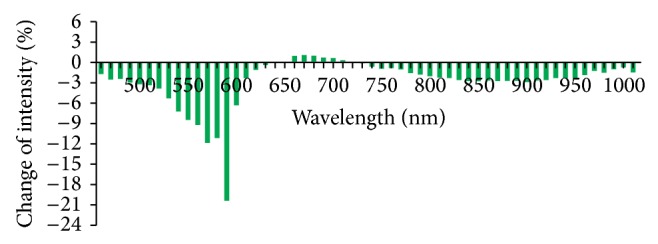
Comparison of rates of reflex intensity change at the PC6 after moxibustion in the Mox group (%, *n* = 10). Note: the number with difference of rates of light reflex intensity at all the wavelengths in this group is great and the rates of light reflex intensity change at 450–640 nm and 720–1000 nm are all negative. The reduction at wave band of 530–590 nm is greatest and the reduction is great at the wave bands of 530–590 nm and is greatest at 580 nm, −20.5%. The rate of light reflex intensity change is increased at the wave band of 600–700 nm, but the increment is less than 5%.

According to analysis ofrate of light reflex intensity change (formula in method 2.6), rates of light reflex intensity change of 56 wavelengths within the range of 450–1000 nm were less than 5% and the rates of light reflex intensity change of 36 wavelengths were less than 1% in the Con group which indicated that the light reflex was stable when there was no stimulation at the PC6 (Figure 7). In the Acu group, except the wavelength of 500 nm, the light reflex intensity was weakened after acupuncture and the rate of light reflex intensity change was all negative. The reduction was great at the wave bands of 540–590 nm and was greatest at 580 nm, −6.5%. The reduction was secondly greatest at 540 nm, −5.2% (Figure 8). In the Mox group, the light reflex intensities at the PC6 were weakened at the wave bands of 450–640 nm and 720–1000 nm and the rate of light reflex intensity change was all negative. The reduction was great at the wave bands of 530–590 nm and was greatest at 580 nm, −20.5%. The rate of light reflex intensity change was increased at the wave band of 600–700 nm, but the increment was all less than 5% (Figure 9). There was overall decline for the rates of light reflex intensity change at the PC6 after acupuncture and moxibustion.

## 4. Discussion 

HSI technique was first applied for remote sensing detection filed and at present it has been widely applied in the fields such as criminal investigation, food safety monitoring, geophysical detection, archaeology, and artistic research preservation [7–9]. With the development of multidisciplinary cross-over studies, HSI technique was gradually applied in medical domain because of its high sensitivity, rich implied information, and safe and noninvasive characteristics. Migita et al. [10] had used the HSI technique to observe the hyperspectral images of the cancer tissues and normal tissues on the corpus linguae flat epithelium in mouse; there was an obvious strong peak at 664 nm in the spectrum of the pathological tissue, whereas the physiological tissue was relatively smooth at this wave peak and the difference between them had also been proved by pathological detection. Gerstner et al. [11] had studied the laryngeal mucosal tumor of the living tissues through combination of HSI technique and endoscope and it was found that there was big difference between the physiological and pathological tissues at the wave bands of 550–590 nm and the physiological and malignant pathologic changes of tissues may be effectively identified at the wave band of 590–680 nm. Some scholars had used this technique for nondestructive examination of the eye diseases and color of iris [12, 13] as well as the clinical evaluation for the treatment process of difficult and complicated disease melanoma and diseases related to the skin and blood vessels [14, 15]. Some scholars [16, 17] had tried to apply the hyperspectral imaging technique in tongue diagnosis studies of traditional Chinese medicine and collect the spectral characteristics at different spectrum wave bands so as to objectify the tongue diagnosis. Application of HSI technique in medical diagnosis and detection is an attempt of a new technique which has practical potential in the medical domain.

The latest HSI technique was used for the first time in the present study to observe the hyperspectral characteristics of the acupoint and acupuncture effects, obtain the hyperspectral characteristics of partial forearm and palmar surface including the PC6 under the normal condition, before and after acupuncture and moxibustion, and learn the strong and weak distribution rule of the spectral reflex intensity of each part. The characteristic change of distinct weakening of the spectral reflex intensity on the skin of the PC6 after moxibustion was observed intuitively and clearly in the hyperspectral images. The research results showed the following: the spectral characteristics at the PC6 were stable under the normal conditions; changes occurred within the wave band of 530–590 nm after acupuncture and moxibustion. In addition, the spectral reflex intensity at the PC6 within this range was reduced after the acupuncture and moxibustion stimulation and the maximum reduction was observed at the wavelength of 580 nm. This result exhibited that the information objects revealed by the spectrum at the wave band of 530–590 nm were changed in the skin tissues of PC6 after acupuncture and moxibustion stimulation and the change of the information objects revealed by the spectrum at wavelength of 580 nm was most significant. It was reported in some studies that the spectral reflex intensity of the skin tissues could reveal change of the blood concentrations in the skin tissue and the spectral reflex intensity of the skin tissues was reduced with increase of the blood concentration in the papilla corium layer [18]. In the blood composition, the spectral absorption peak of hemoglobin appeared at about 580 nm [19]. Was the significant change which appeared at the wave band of about 580 nm in the curve of spectrum of the skin tissue at the PC6 after Acu-mox stimulation related to the change of the hemoglobin content in the acupoint area? It was provided in the previous studies of our laboratory that Acu-mox stimulation could significantly improve the blood microcirculation perfusion of local acupoint, adjacent acupoint area, and the related organ tissues [20–22]. Therefore we considered that the blood perfusion at the acupoint area and hemoglobin content in the corium layer of the tissue were increased after acupuncture and moxibustion and the increased absorption for the light at wavelength of 580 nm may be the reason for decline of the spectrum curves at the wave band of 530–590 nm. It could be seen that the spectral change after moxibustion was obviously greater than that after acupuncture which was identical with the result that the blood perfusion increase of acupoint area after moxibustion at the PC6 was greater than the result of acupuncture [21, 22]. There was correspondence between the two results which revealed that effects of moxibustion in terms of these two aspects were better than acupuncture.

The light reflex-absorption characteristics of the acupoint skin tissue are revealed by the spectral characteristic of the tissue which may be related to the factors such as functional activities (temperature, microcirculation, material metabolism, etc.) of local tissue and function of the skin tissue structure (texture, sweat gland, and sebaceous gland). The change characteristics of the PC6 at the wave band of 530–590 nm were observed in the study. Whether they were also related to other functions or structures of the local tissue such as density degree of the skin cells and amount of the interstitial fluid beside the elevation of hemoglobin content at the acupoint promoted by Acu-mox stimulation should be further discussed in combination with histomorphological, biochemical, and other biological imaging methods, to find the functional, material, and structural bases of the spectral characteristic.

## 5. Conclusion 

The color change of the acupoint area before and after acupuncture and moxibustion was clearly and intuitively exhibited by the hyperspectral images. The characteristic wavelength of the spectrum curves at the PC6 after acupuncture and moxibustion stimulation was exhibited at 530–590 nm. The results for the observation of acupuncture and moxibustion effects in this study have revealed the triple information such as space, spectrum, and radiation of the substance being examined by HSI technique and the advantages of the technique for positioning and quantitative analysis. Therefore, the technique may provide a new direction for further study of Acu-mox effects. In general, HSI technique can detect the biological information of tissue to provide scientific evidence for clinical and scientific research. It will certainly become an important means for the medical study in the future. Our laboratory has used this latest imaging technique for the first time to observe the HSI characteristics of the acupoint and Acu-mox effects and substantiated the contents of effects of Acu-mox in terms of spectral characteristics. At the same time this study is also an innovative attempt for the Acu-mox mechanism and acupoint characteristic study using the newest technique method.

## Supplementary Material

Hyperspectral imaging (HIS) technique is an image data technique which has beendeveloped in recent years which integrated the advanced technologies from the fieldssuch as optics, optoelectronics, electronics, information processing, computer scienceand so on and it is an emerging technology withintegration of traditionaltwo-dimension imaging technology and spectrumtechnology. Application ofhyperspectral imaging technique is justbeginning in the medical domain and there isno report of acupuncture study. This technique is used for preliminary observation andanalysis for the spectral changes of the acupoint area before and after acupuncture inthis manuscript so as to learn the possibility of using the technique in theacupuncture, main and collateral channels and acupoint studies and lay the foundationfor exploration of new research fields for Acu-mox studies. In this manuscript, thistechnique is used for the first time and some regular and characteristicspectral changes after acupuncture and moxibustion have been obtained.
