# Supplementary material for: A Pilot Study on Effects of Acupuncture and Moxibustion by Hyperspectral Imaging Technique
Source: Evid Based Complement Alternat Med. 2014 Dec 3;2014:135212. doi: 10.1155/2014/135212 (PMC4269316; doi:10.1155/2014/135212)
Supplement: Supplementary file 1 — Hyperspectral imaging (HIS) technique is an image data technique which has beendeveloped in recent years which integrated the advanced technologies from the fieldssuch as optics, optoelectronics, electronics, information processing, computer scienceand so on and it is an emerging technology withintegration of traditionaltwo-dimension imaging technology and spectrumtechnology. Application ofhyperspectral imaging technique is justbeginning in the medical domain and there isno report of acupuncture study. This technique is used for preliminary observation andanalysis for the spectral changes of the acupoint area before and after acupuncture inthis manuscript so as to learn the possibility of using the technique in theacupuncture, main and collateral channels and acupoint studies and lay the foundationfor exploration of new research fields for Acu-mox studies. In this manuscript, thistechnique is used for the first time and some regular and characteristicspectral changes after acupuncture and moxibustion have been obtained. [file 135212.f1.pdf]

Figures and Notes

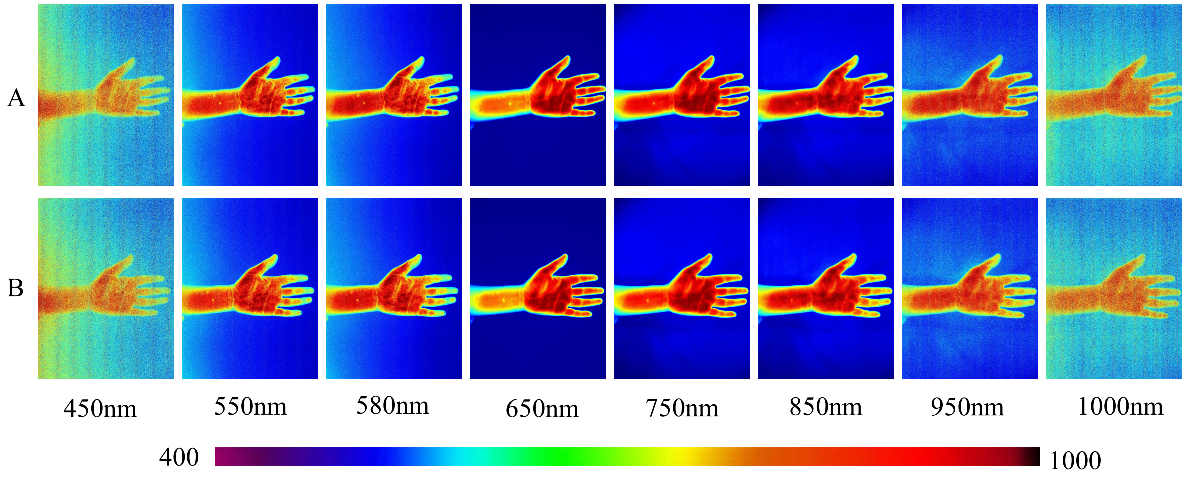

Figure 1 Hyperspectral Images of the Forearm and Palm at Different Wavelengths at 0 and 10 min of the Con Group

Note: The images of forearm and palm which are normal condition at 0 min(A) and 10 min(B) are shown for selected wavelengths from 450,550,580,650,750,850,950 and 1000nm. The color scale indicates the degree of light reflex intensity and the intensity from left to right is from weak to strong. The colors of the thenar eminence, antithenar eminence and 2/3 part in the centre of the palm closely adjacent to antithenar eminence are darkest and the light reflex is strongest. The small part of the centre of the palm adjacent to thenar eminence is lighter and the light reflex is weaker. There is no significant change for the color distribution of the spectral images of PC6 and other areas on the hand between 0 min(A) and 10 min(B).

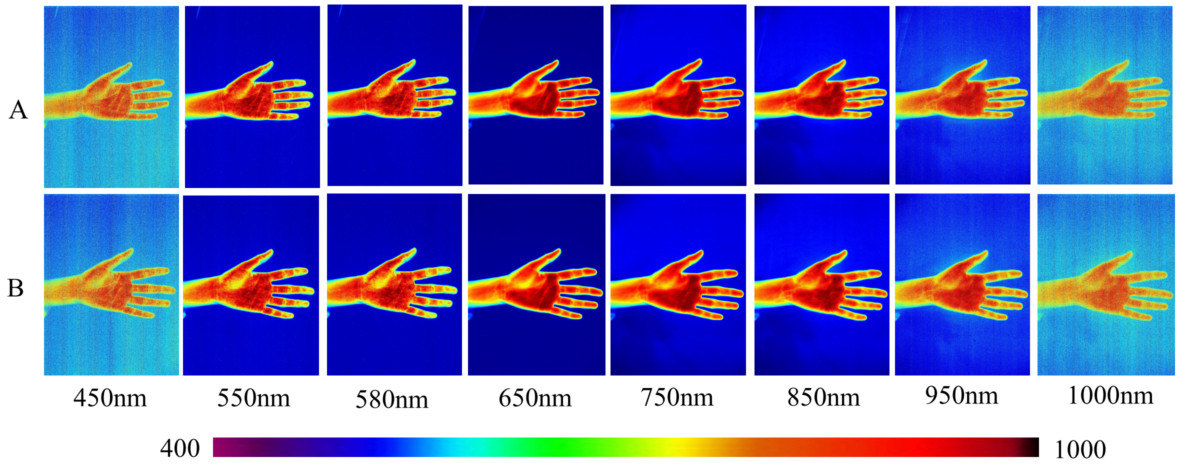

Figure 2 Hyperspectral Images of the Forearm and Palm at Different Wavelengths at 0 and 10 min

Note: The images of forearm and palm which are at 0 min(A) and 10 min(B) of the Acu group are shown for selected wavelengths from 450,550,580,650,750,850,950 and 1000nm. In Figure 2(A), there is no significant change for the color distribution of the spectral images of PC6 and other areas on the hands compared to Figure 1(A). According to the color scale, in Figure 2(B), there is left shift of color at the corresponding wavelength points compared to Figure 2(A) which indicates that the light reflex intensity is weakened after acupuncture.

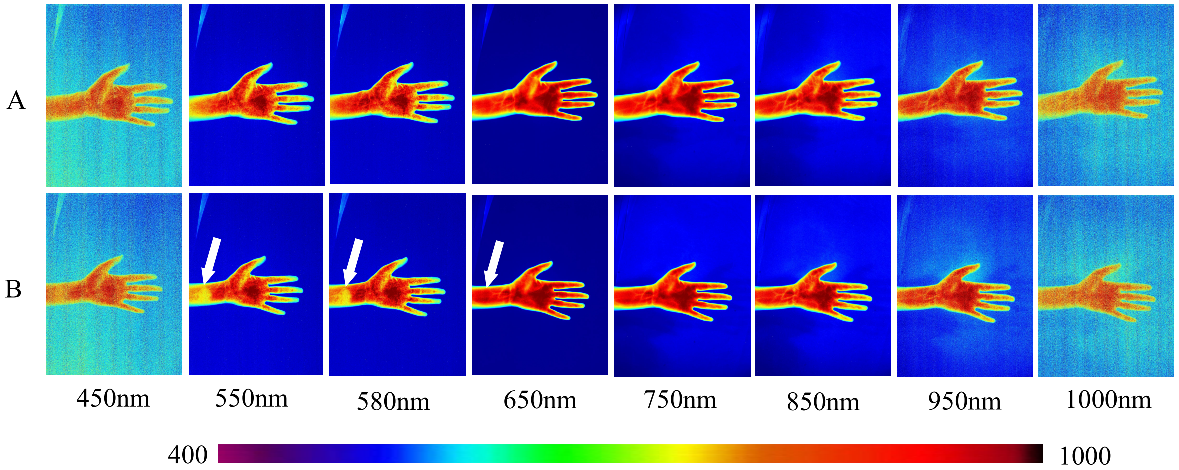

Figure 3 Hyperspectral Images of the Forearm and Palm at Different Wavelengths at 0 and 10 min of the Mox Group

The images of forearm and palm which are at 0 min(A) and 10 min(B) of the Mox group are shown for selected wavelengths from 450,550,580,650,750,850,950 and 1000nm. In Figure 3(A), there is no significant change for the color distribution of the spectral images of PC6 and other areas on the hands compared to Figure 1(A). According to the color scale, in Figure 3(B), the left shift of color is significant at the corresponding wavelength points compared to Figure 3(A) which indicates that the light reflex intensity is significantly weakened after moxibustion. The color change is most significant at the PC6 (indicated by the arrow) and colors of other areas of hands are also changed.

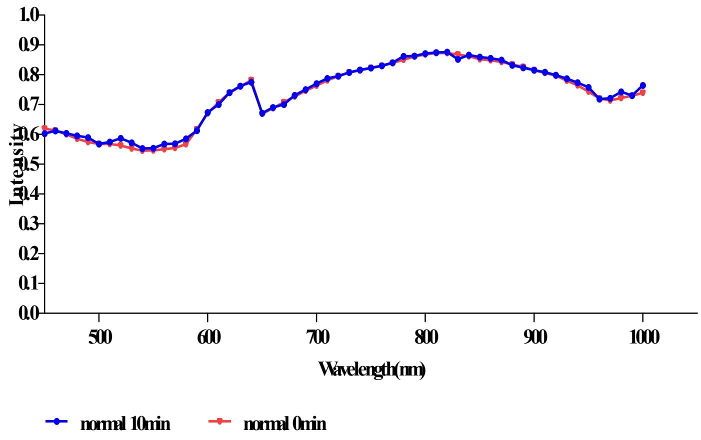

Figure 4 Comparison of the Light Reflex Intensity Values of PC6 before and after 10 min in the Con Group (Mean, n =10)

Note: The distribution mean values of curves at all the Wavelengths at 0 min basically overlap those at 10 min which indicates that there is no significant change of light reflex intensity before and after 10 min.

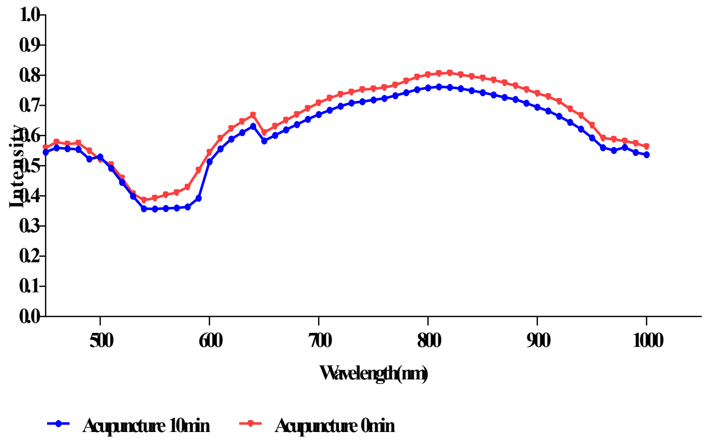

Figure 5 Comparison of the Light Reflex Intensity Values of PC6 before and after acupuncture in the Acu Group (Mean, n =10)

Note: The distribution mean values of curves at all the wavelength are different after 510 nm before and after acupuncture and the values after acupuncture are all lower than those before acupuncture which indicates that acupuncture leads to reduction of light reflex intensity.

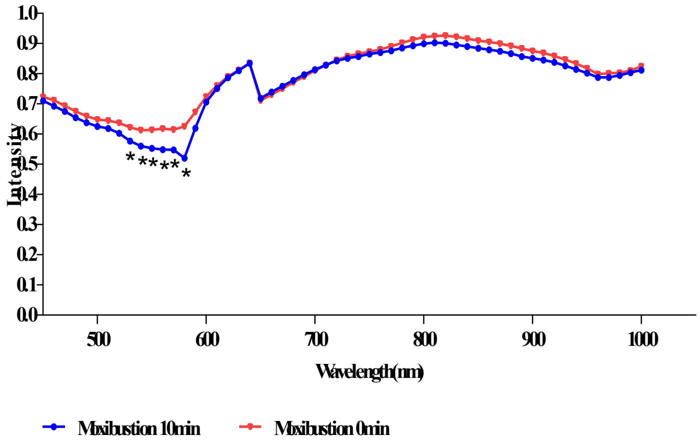

Figure 6 Comparison of the Reflex Intensity Values of PC6 before and after Moxibustion in the Mox Group (Mean, n=10)

Note: There is significant difference at Wave band of 530-590 nm and wave band of 780-960 nm before and after the moxibustion, the values after moxibustion are lower than those before moxibustion which are especially significant at six points between 540 nm and 590 nm. There is statistical difference and $^{*}p<0.05$.

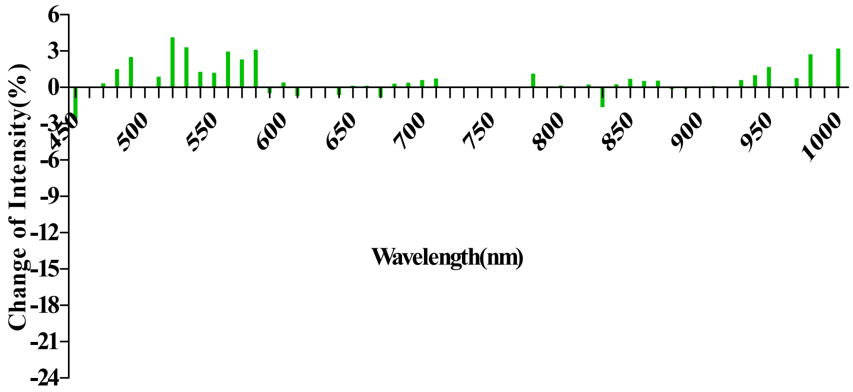

Figure 7 Comparison of Rates of Reflex Intensity Change at the PC6 after 10 min in the Con Group (%, n = 10)

Note: The rates of reflex intensity change of all the Wavelengths are all lower than 5%.

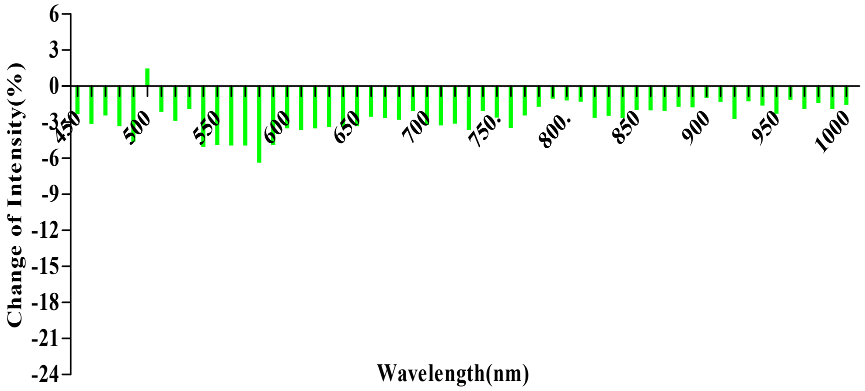

Figure 8 Comparison of Rates of Reflex Intensity Change at the PC6 after Acupuncture in the Acu Group (%, n = 10)

Note: The rates of light reflex intensity change at all the wave bands are all negative. The reduction is greatest at 580 nm, -6.5%.

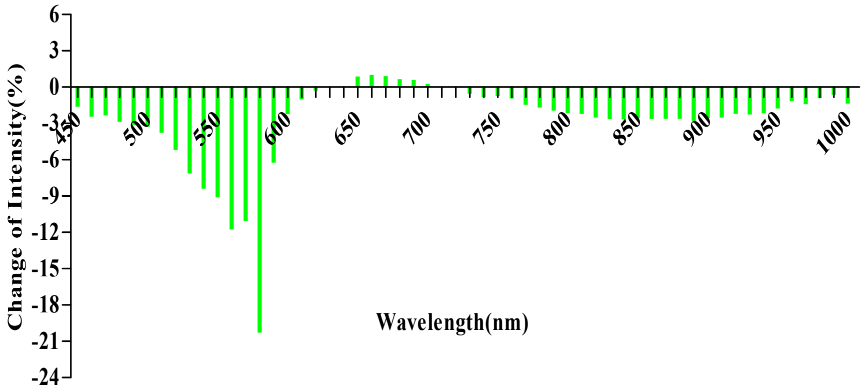

Figure 9 Comparison of Rates of Reflex Intensity Change at the PC6 after Moxibustion in the Mox Group (%, n = 10)

Note: The number with difference of rates of light reflex intensity at all the Wavelengths in this group is great and the rates of light reflex intensity change at 450-640nm and 720-1000nm are all negative. The reduction at wave band of 530-590 nm is greatest and

The reduction is great at the wave bands of 530-590 nm and is greatest at 580 nm, -20.5%. The rate of light reflex intensity change is increased at the wave band of 600-700 nm, but the increment is less than 5%.
